# Induced Tamoxifen Resistance is Mediated by Increased Methylation of E-Cadherin in Estrogen Receptor-Expressing Breast Cancer Cells

**DOI:** 10.1038/s41598-019-50749-1

**Published:** 2019-10-02

**Authors:** Qi Wang, Melisa Gun, Xing-yu Hong

**Affiliations:** 1 Department of Emergency, The First Affiliated Hospital of Zhengzhou University, Zhengzhou City, Henan Province 450002 China; 2 Medipol University, Istanbul, Turkey; 3 Department of Surgery, China-Japan Union Hospital of Jilin University, Changchun, 130031 China

**Keywords:** Diagnostic markers, Breast cancer

## Abstract

Estrogen receptor-positive breast cancers are treated with tamoxifen, a drug that competitively inhibits the binding of estrogen to its receptor. Resistance to tamoxifen is a major hurdle in effective management of target breast cancer patient population. A number of dynamic changes within the tumor microenvironment, including the phenomenon of epithelial to mesenchymal transition (EMT), determine the response to endocrine therapy. EMT is marked by silencing or suppression of epithelial marker, E-Cadherin and we found significantly down-regulated E-Cadherin, among other epithelial markers, and a significantly up-regulated mesenchymal marker, Twist, among other mesenchymal markers, in a model system that comprised of tamoxifen sensitive MCF-7 cells and their tamoxifen-resistant counterparts, MCF-7-TAM, developed by chronic and escalating exposure of parental cells to tamoxifen. Further, E-cadherin, but not Twist, was differentially expressed in MCF-7-TAM cells because of differential methylation. Treatment with demethylating agent 5-azacytidine increased the expression of E-cadherin thus verifying a role of methylation in its silencing and, moreover, 5-azacytidine treatment also re-sensitized MCF-7-TAM cells to tamoxifen, as evaluated by assays for viability, apoptosis and migration potential. The 5-azacytidine effects were similar to effects of E-cadherin overexpression in MCF-7-TAM cells. This work describes novel mechanism of E-cadherin downregulation in tamoxifen resistant breast cancer cells. Further studies are needed to exploit this information for betterment of breast cancer therapy.

## Introduction

Breast cancer is a cancer with many subtypes and estrogen receptor (ER)-positive breast cancers, which constitute about 70% of all breast cancers, are treated with tamoxifen therapy^1^. Tamoxifen binds to estrogen receptor, but does not activate it^2^, rendering a good inhibition of estrogen receptor-mediated signaling. Target patients experience the benefits of tamoxifen resistance initially but a large number of patients eventually start showing signs of resistance which remains a major hurdle in the effective management of breast cancer^3^. Because of the heightened interest in breast cancer, particularly the estrogen receptor-positive subtype, a number of investigations have been carried out to understand the basis of tamoxifen resistance and a number of genes, factors and signaling pathways are now believed to contribute to resistance to tamoxifen^4–8^. Even though so many possible mechanisms have been proposed and investigated, the benefits in clinics are minimal.

Epithelial to mesenchymal transition (EMT) has been target of research in the fight against drug resistance in breast cancer for several years^9–12^. Even the phenomenon of resistance to tamoxifen in estrogen receptor positive breast cancer cells is believed to involve EMT as one of the mechanism^13–17^. EMT fundamentally involves a transition from more organized and robust epithelial to a considerably more motile mesenchymal phenotype. The process of EMT is marked by decreased expression of epithelial markers and an enrichment of mesenchymal markers and thus relative expression of these markers serves as a good tool to evaluate EMT. E-cadherin is an important epithelial marker that is down-regulated during the progression of EMT. E-cadherin can convert to N-cadherin and such ‘cadherin switch’ is often seen at the time of cancer progression resulting in aggressive behavior and increased metastases^18^. Even though E-cadherin is often evaluated with respect to its relative expression in cells undergoing EMT, the mechanistic details of down-regulation of E-cadherin, particularly its epigenetic regulation, if any, and in the context of resistance against tamoxifen are totally unknown.

## Material and Methods

### Cell culture and other reagents

MCF-7 (ATCC HTB-22) cells were obtained from Beijing Zhongyuan Limited (official ATCC distributors; Beijing, China) and MCF-7-TAM were generated by exposing MCF-7 cells to increasing concentrations of tamoxifen over a period of 6 months starting with a dose equivalent to IC-5 of tamoxifen. Cells were maintained in the culture medium and passaged, as per ATCC’s instructions. All cells were cultured in 5% CO_2_ humidified atmosphere at 37 °C within our laboratory. Tamoxifen and 5-azacytidine were purchased from Sigma Chemical Company (Shanghai, China).

### MTS assay

We performed MTS (3-(4,5-dimethylthiazol-2-yl)-5-(3-carboxymethoxyphenyl)-2-(4-sulfophenyl)-2H-tetrazolium) assay to evaluate cell viability. The assay was done in 96-well plates and cells were seeded atleast 24 hours prior to the assay. Individual treatments were performed, as indicated. At the end of treatment, absorbance was measured on a Shimadzu spectrophotometer (Japan).

### Apoptosis detection

Cells were treated with increasing concentrations of tamoxifen, as indicated for individual figures, and harvested, fixed, and labeled exactly according to the instructions provided with the TiterTACS Kit (R&D Systems, Shanghai, China). Colorimetric readings were recorded on a Shimadzu spectrophotometer (Japan).

### Measurement of migration of cancer cells

We used the Millipore QCM 5 µm Chemotaxis Assay 24-well-colorimetric kit (Shanghai, China) which provides a quick and efficient system for quantitative determination of cell migration after cells are subjected to treatments. The Millipore 5 µm QCM Chemotaxis Assay 24-well-colorimetric is performed in a Migration Chamber, based on the Boyden chamber principle. The 5 µm pore size of this assay′s Boyden chambers is appropriate for studying a subset of fibroblast or cancer cell migration. In our experiments, cells were first subjected to drug treatment, trypsinized, counted and seeded in equal numbers on the provided 24-well plates. Assays were performed exactly as suggested by the Instructions. Colorimetric readings were recorded on a Shimadzu spectrophotometer (Japan).

### Quantitative RT-PCR

RNA was extracted from cells using Trizol (Thermo Fisher Scientific, Shanghai, China) and quantitative RT-PCR was performed as described earlier^19^. cDNA synthesis was carried out with SuperScript III reverse transcription (Thermo Fisher Scientific, Shanghai, China) and the quantitative real-time PCR performed using iTaq Universal SYBR Green Supermix (Bio-Rad, China). The mRNA expression levels of were quantified by measuring the threshold cycle (Ct).

### E-cadherin over-expression

E-cadherin, cloned in pCMV6, was obtained from Origene (Beijing, China). Transfection of cloned E-cadherin into MCF-7TR cells was done using lipofectamine 2000 reagent (Thermo Fisher Scientific, Shanghai, China), following the procedure suggested by the company.

### Promoter methylation assays

We studied the methylation of individual genes, using MethylDetector Bisulfite conversion kit (Active Motif, China) which involved heat denaturation in the presence of the bisulfite conversion reagent. All the steps were followed, as described in the Instructions, including PCR.

### Statistical analysis

Each experiment was performed a minimum of three times and Student t test (unpaired, one-tailed) was employed to compare individual test groups with the respective controls. Significant difference was set at 0.05.

## Results

### Characterization of resistant cells

**Figure 1:**
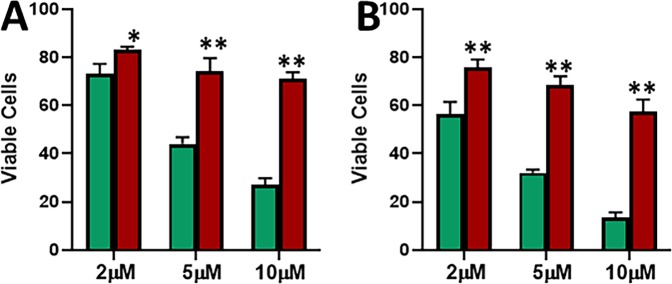
Characterization of tamoxifen resistance. Parental MCF-7 (green) and tamoxifen-resistant MCF-7 cells (MCF-7-TAM; red) were treated with indicated concentrations of tamoxifen for 48 **(A)** or 72 **(B)** hours. Viable cells were quantitated using MTS assay. *p < 0.05, **p < 0.01.

MCF-7 cells chronically treated with tamoxifen eventually developed resistance against the drug. This resistance was confirmed by cell viability MTS assay. As shown in Fig. 1A, when MCF-7 cells were treated with increasing concentrations of tamoxifen for a time duration of 48 hours, a dose dependent decrease in cell viability was observed. Even the resistant cells showed a decreasing trend but the cells were significantly more viable than the parental MCF-7 cells at all the three doses – 2 μM, 5 μM and 10 μM. Additionally, we prolonged the treatment time and now exposed the cells to tamoxifen for 72 hours and still found the same trend (Fig. 1B). There still was a dose-dependent decrease in viability of parental MCF-7 and tamoxifen resistant MCF-7-TAM cells, but the MCF-7-TAM cells were significantly (p < 0.01) more resistant than the parental cells. Thus, we found through dose-and time-dependent analyses that MCF-7-TAM cells were considerably more resistant to tamoxifen than the parental cells, and were thus an excellent model to study the mechanism of induced tamoxifen resistance.

### Tamoxifen-resistant cells exhibit reduced apoptosis and increased migration

**Figure 2:**
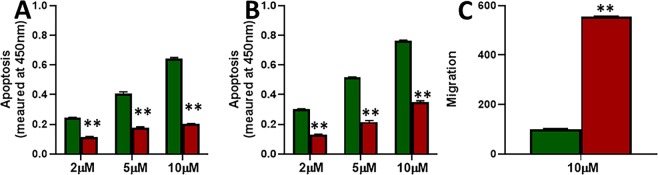
Induction of apoptosis by tamoxifen and effects on migration. Parental MCF-7 (green) and tamoxifen-resistant MCF-7-TAM cells (red) were treated with indicated concentrations of tamoxifen for 48 **(A)** or 72 **(B)** hours and then the apoptosis was detected. **(C)** Migration of MCF-7 and MCF-7-TAM cells was evaluated after cells had been subjected to 10 μM tamoxifen for 48 hours. **p < 0.01.

Chemotherapeutic drugs mostly function through induction of apoptosis and such action also results in reduced migratory abilities, therefore, we checked the induction of apoptosis by tamoxifen and the resulting affect on migration in our cell line models. As shown in Fig. 2A,B, tamoxifen doses induced a dose-dependent as well as time-dependent increase in apoptosis in both the cells. However, the induction of apoptosis in MCF-7-TAM cells was significantly (p < 0.01) less, compared to the parental MCF-7 cells. Even when tested for migration after treatment with a 10 μM dose for 48 hours, MCF-7-TAM cells still retained significantly (p < 0.01) more migratory potential that the parental MCF-7 cells. Thus, tamoxifen induced significantly more apoptosis in parental MCF-7 cells and also reduced the migratory potential of these cells, compared to the tamoxifen-resistant cells.

### Evaluation of EMT

**Figure 3:**
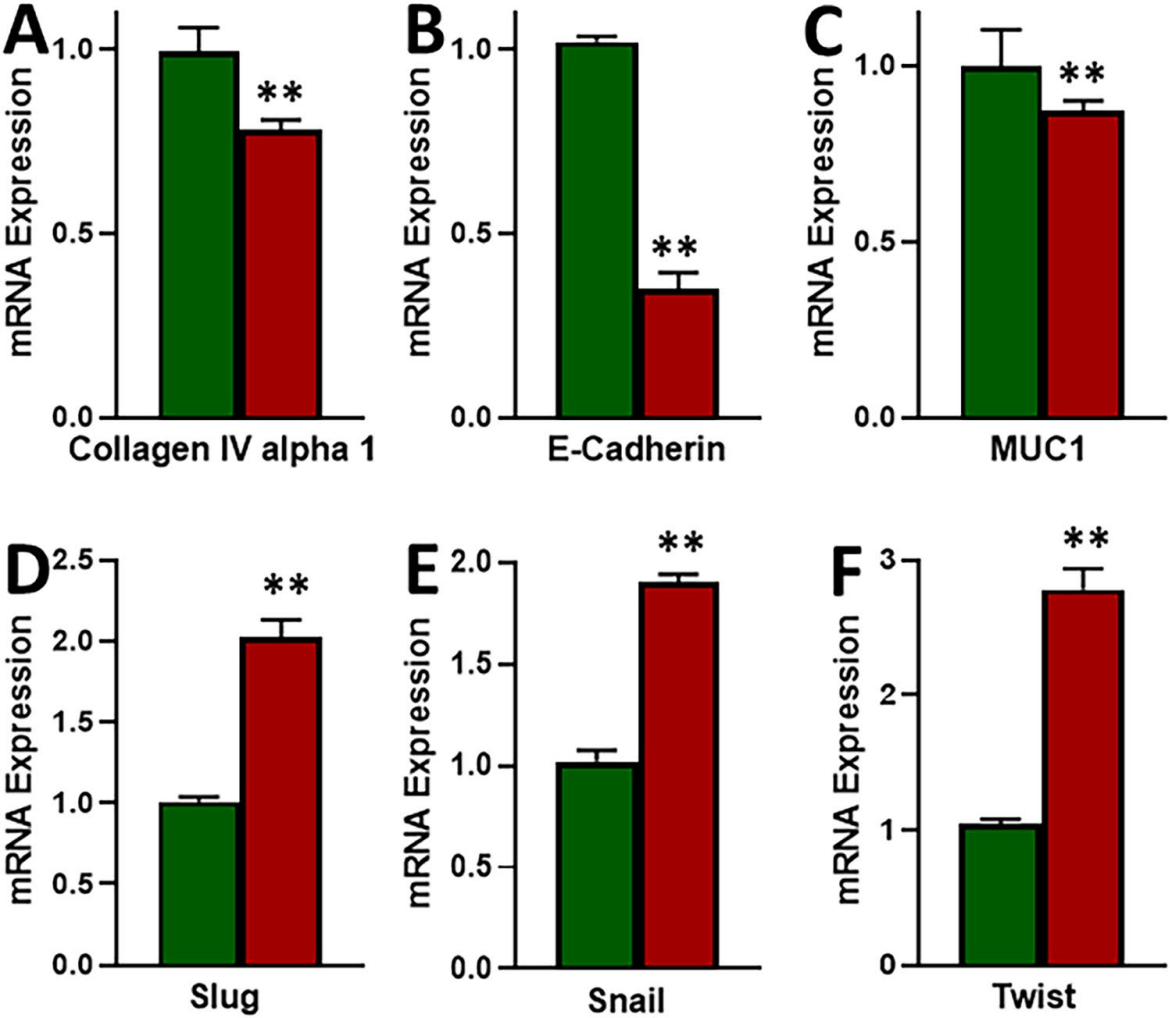
Induction of EMT in tamoxifen-resistant cells. (**A**–**C)** Epithelial markers and **(D**–**F)** Mesenchymal markers were evaluated by quantitative qRT-PCR in parental MCF-7 (green) and tamoxifen-resistant MCF-7-TAM (red) cells. GAPDH was used as internal control during the PCR. **p < 0.01.

EMT has been proposed to be a major factor affecting the response of cancer cells to therapy, therefore, we evaluated EMT in our cell line models. We tested three epithelial markers (Collagen IV alpha 1, E-cadherin and MUC1) and three mesenchymal markers (Slug, Snail and Twist). As shown in Fig. 3, we found decreased expression of all the three epithelial markers and the increased expression of all the three mesenchymal markers in MCF-7-TAM cells, as compared to the parental MCF-7 cells. In our results, we found E-cadherin to be the most affected epithelial marker and Twist to be the most affected mesenchymal marker. Thus, we found elevated mesenchymal markers and decreased epithelial markers in tamoxifen-resistant cells which means that EMT was induced in these cells.

### E-cadherin is epigenetically down-regulated

**Figure 4:**
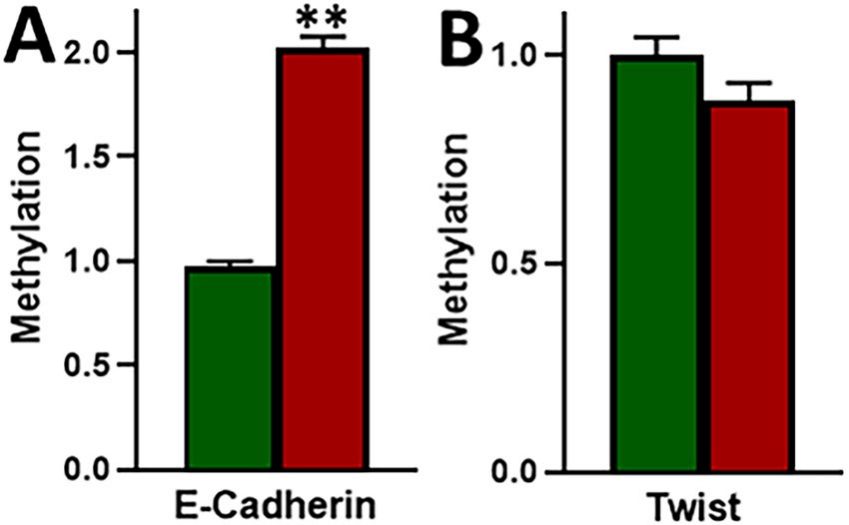
Methylation of genes. Methylation of promoter regions of **(A)** E-cadherin and **(B)** Twist was evaluated in parental MCF-7 (green) and tamoxifen-resistant MCF-7-TAM (red) cells and the methylation in MCF-7-TAM is shown, relative to methylation in MCF-7 cells. **p < 0.01.

We evaluated methylation as a possible mechanism for the down-regulation of E-cadherin and the up-regulation of Twist, the two most differentially expressed EMT markers revealed in the last figure. We found significantly increased methylation of E-cadherin promoter (Fig. 4A) in tamoxifen-resistant MCF-7-TAM cells, compared to the parental MCF-7 cells. Further, even though the methylation of Twist was relatively low in MCF-7-TAM cells, compared to MCF-7 cells, the levels were not determined to be significantly different.

### Effect of E-cadherin overexpression

**Figure 5:**
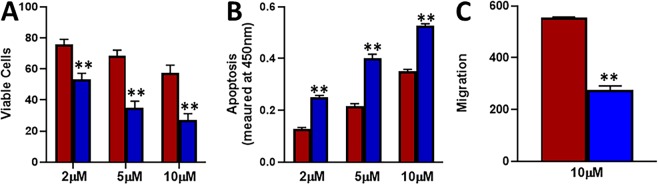
Effect of E-cadherin overexpression. E-cadherin was over expressed, by transfection of plasmid carrying the construct, in MCF-7-TAM cells. Tamoxifen-resistant MCF-7-TAM (red) cells and the tamoxifen-resistant MCF-7-TAM with over-expressed E-cadherin (blue) were treated with indicated concentrations of tamoxifen for 72 hours **(A)**. Viable cells were quantitated using MTS assay. **(B)** Apoptosis was also detected in these cells after treatment with tamoxifen for 72 hours. **(C)** Migration was assessed after cells had been subjected to 10 μM tamoxifen for 48 hours. **p < 0.01.

In our evaluation of EMT markers shown above, we found down-regulation of E-cadherin. Therefore, we overexpressed E-cadherin in tamoxifen-resistant MCF-7-TAM cells and tried to evaluate if restoration of E-cadherin could reverse the action of tamoxifen. As shown in Fig. 5A, we found that over-expression of E-cadherin significantly (p < 0.01) reduced the viability of MCF-7-TAM cells at all the three doses – 2 μM, 5 μM and 10 μM, when cells were treated with tamoxifen for 72 hours. At the same time point, cells with over-expressed E-cadherin underwent significantly (p < 0.01) more apoptosis (Fig. 5B). Further, migration potential of cells was significantly decreased (p < 0.01) (Fig. 5C). Thus, we found that over-expression of E-cadherin significantly sensitized the estrogen receptor-positive breast cancer cells to tamoxifen and tamoxifen was found to reduce viability/migration and increase apoptosis in these cells.

### Effect of treatment with 5-azacytidine

**Figure 6:**
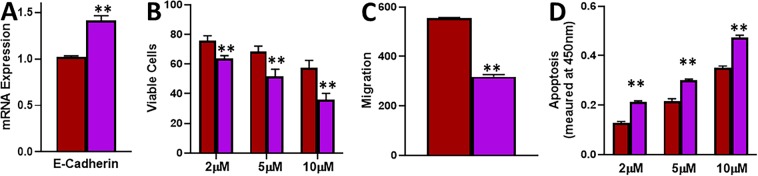
Effect of 5-azacytidine. (**A)** MCF-7-TAM cells were exposed to 5-azacytidine and the effect on E-cadherin’s mRNA expression was evaluated in MCF-7-Tam cells treated with 5-azacytidine (purple), relative to MCF-7-TAM cells (red), by quantitative qRT-PCR. GAPDH was used as internal control. **(B)** Cells were treated with indicated concentrations of tamoxifen for 72 hours and viability was evaluated by MTS assay. **(C)** Cells were also treated with 10 μM tamoxifen for 48 hours and the resulting effects on migration potential evaluated. **(D)** Apoptosis was also detected in these cells after treatment with tamoxifen for 72 hours. **p < 0.01.

In the results described above, we observed increased methylation of E-cadherin in tamoxifen resistant cells. Therefore, we wanted to test if this was mechanistically linked to tamoxifen-mediated effects. We used 5-azacytidine as an agent that demethylates DNA. We chronically treated MCF-7-TAM cells with 1 μM 5-azacytidine for 6 cell passages and found that the expression of E-cadherin was increased after treatment with 5-azacytidine (Fig. 6A). As shown in Fig. 6B–D, this treatment also resulted in decreased viability of cells, a decrease in migration potential and an increase in the apoptosis. Thus, 5-azacytidine treatment also increased the sensitivity of tamoxifen-resistant cells to tamoxifen.

## Discussion

Breast cancer remains a major killer among the females^20–22^. Treatment of breast cancer patients depends upon factors such as presence of estrogen receptor, progesterone receptor or HER2 overexpression. Presence of one or more of these receptors makes it convenient to enroll the patients for targeted therapy. Endocrine therapy, such as tamoxifen, is effective in breast cancer patients with overexpression of estrogen receptor. Tamoxifen functions via binding to estrogen receptor and competitively inhibiting the binding of estrogen to its receptor. It does not affect the levels of circulating estrogen^3^.

In our analyses, we found significantly higher methylation of E-cadherin promoter in tamoxifen-resistant cells. This can clearly be a reason for its observed down-regulation because methylation of gene promoters leads to their silencing^23^. Now, that does not mean that this is the only mechanism and certainly more mechanism(s) cannot be ruled out, particularly when we look at the relative expression of the most differentially expressed mesenchymal marker Twist. Twist was found to be highly elevated in tamoxifen resistant cells, however, even though the methylation of its promoter was slightly reduces, as would be expected in cells undergoing EMT, the degree of reduced methylation was not significant enough. This would mean that up-regulation of Twist is because of some other mechanism that does not necessarily involves methylation. On the other hand, the levels of methylation were found to be significantly more for E-cadherin, giving confidence in pointing out some epigenetic regulation. This was further confirmed by experiment using 5-azacytidine, which is a demethylating agent^24^ and, by definition, reduces methylation by removing methyl groups. When we treated cells with 5-azacytidine, not only the suppression of E-cadherin inhibited in tamoxifen-resistant cells, but the resulting attenuation of tamoxifen effects, such as on cellular apoptosis and migration potential, was also observed which clearly gives weightage to the methylation of E-cadherin as a principal mechanism causing its down-regulation in the tamoxifen-resistant cells.

Epigenetic regulation of tumor suppressor genes has been realized for quite some time now^25–27^ and the functional implications of these epigenetic events are coming to light now. The epigenetic changes within the tumor microenvironment assume further significance^28^ where so many cell types mutually interact within a small space with many of them manipulated into serving the needs of cancer cells. Any changes in the expression of factors within the tumor microenvironment are thus bound to have profound effect on cellular signaling and the growth, proliferation, invasion, metastasis and angiogenesis of cancer cells because they happen inside or within a close vicinity of cancer cells. Further, E-cadherin has long been identified as a prominent biomarker with critical role in cancer progression. It has even been called a ‘gatekeeper’ to breast cancer tumorigenesis^29^ and although there exists plethora of information on its up- or down-regulation under different conditions, the mechanisms remain almost unknown. Thus, keeping in view such gap in our information, our present study provides some novel information on the regulation of E-cadherin, particularly in breast cancers that have turned refractory to endocrine treatment. While we provide *in vitro* evidence in support of a novel mechanism of regulation of E-cadherin in tamoxifen-resistant breast cancer, we understand that further animal studies are needed to validate these findings so that the information could be of use in future clinical trials.

Even though no specific guidelines were recommended for inclusion of EMT markers in clinical studies until a few years back, this has considerably changed now. New data supports an evaluation of EMT markers, even in relation to novel targets such as circulating tumor cells^30,31^. Moreover, the small clinical study on 22 patients has suggested benefit of this practice even in the determination of response to a chemotherapy^31^. Further, epigenetics is now understood to play big role in determination of drug sensitivity in breast cancer^32^, even in the delicate balance between resistance and sensitivity to tamoxifen^33–36^. This not only suggests that our work is timely, but also calls for further studies to improve our understanding of the underlying mechanisms, which can lead to much improved targeted therapies for the treatment of estrogen receptor-expressing breast cancers.

## Data Availability

All the authors declare that all data from the study is reported in this article.
